# A bibliometric analysis of minimally invasive esophagectomy for esophageal cancer (2005–2024): mapping the knowledge landscape and future trends

**DOI:** 10.1007/s00464-026-12824-3

**Published:** 2026-04-30

**Authors:** Wei He, Guangliang Qiang

**Affiliations:** Department of Thoracic Surgery, Peking University Third Hospital, Beijing, China

**Keywords:** Bibliometric, Minimally invasive esophagectomy, Esophageal cancer, Bibiometrix, VOSviewers, Citespace

## Abstract

**Background:**

Minimally invasive esophagectomy (MIE) has become central to the surgical management of esophageal cancer, yet a comprehensive bibliometric overview of this evolving field is lacking. This study aims to map the research landscape, identify key contributors and trends, and forecast future directions in MIE.

**Methods:**

A literature search was conducted in the Web of Science Core Collection (WoSCC) for publications from 2005 to 2024. Bibliometric analysis was performed using Bibliometrix (via Biblioshiny), VOSviewer, and CiteSpace to analyze publication trends, collaborations, influential entities, and keyword.

**Results:**

We identified 1427 English articles and reviews. Annual publications peaked in 2022. China, Japan, the USA, and the Netherlands were the most productive countries, with the Netherlands achieving the highest citation impact. Collaborative networks were primarily intra-national. *Surgical Endoscopy and Other Interventional Techniques* and *Diseases of the Esophagus* were core journals, while seminal RCTs appeared in high-impact journals like *The Lancet*. Keyword analysis revealed four major clusters: Robot-assisted MIE (RAMIE), thoracoscoscopic-laparoscopic esophagectomy, neoadjuvant therapy for squamous cell carcinoma, and the Ivor Lewis technique. Research trends evolved from techniques/complications to neoadjuvant and immunotherapy for esophageal squamous cell carcinoma.

**Conclusions:**

This first comprehensive bibliometric study of MIE confirms its established status, with RAMIE and immunotherapy as current hotspots. Future advancement hinges on large-scale, multinational RCTs and long-term outcome studies. This analysis provides a foundational map to guide research priorities and foster global collaboration.

Esophageal cancer represents a major global oncologic challenge. Epidemiological data from 2020 positions it as the seventh most commonly diagnosed cancer (604,000 new cases) and the sixth leading cause of cancer death (544,000 deaths), responsible for about one in every 18 cancer fatalities worldwide.

The epidemiology is intricately linked to histologic subtype, with significant geographic disparities. Squamous cell carcinoma (SCC) predominates in high-risk regions of Asia, while adenocarcinoma (AC) accounts for approximately two-thirds of cases in high-income nations, where risk factors such as obesity, gastroesophageal reflux, and Barrett's esophagus are prevalent [1].

For locally advanced esophageal cancer, surgery remains the primary curative modality. Esophagectomy with lymphadenectomy is standard for T2N0M0 disease, whereas preoperative therapy (chemotherapy or chemoradiotherapy) is recommended for more advanced stages to improve outcomes [2]. Over recent decades, minimally invasive esophagectomy (MIE) has become central to surgical practice. The combined thoracoscopic-laparoscopic technique underpins common procedures like Mckeown and Ivor Lewis esophagectomy. Alternative approaches, such as transhiatal (with or without transcervical) esophagectomy, are also actively researched. The advent of robot-assisted MIE (RAMIE) further expands the technical repertoire [3]. Across all approaches, achieving procedural efficiency and minimizing complications, such as those benchmarked in recent studies [4], are paramount goals.

Therapeutic strategies continue to evolve beyond surgery alone. Neoadjuvant regimens, including chemoradiotherapy and the rapidly emerging immunotherapy [5], are integrated to enhance resection rates and prognosis.

In this complex and evolving research landscape, bibliometric analysis offers a powerful tool to quantitatively assess scientific output, map knowledge structures, and identify trends [6]. It provides a comprehensive overview of a field's development, highlights key contributors and collaborative networks, and helps anticipate future research directions [7]. Such analysis is invaluable for guiding research priorities and resource allocation [8].

Despite the wealth of clinical research on MIE for esophageal cancer, a comprehensive bibliometric synthesis is lacking. Given the diversity of surgical techniques and their integration with multimodal therapies, a systematic analysis is needed to clarify the current state, hotspots, and collaborative patterns in the field. Therefore, this study aims to conduct a bibliometric analysis to delineate the knowledge landscape, trace evolutionary trends, and identify frontiers in MIE research, thereby offering evidence-based guidance for future scientific inquiry and clinical innovation.

## Method

### Search strategy

Web of science (WOS) is one of the most commonly used academic database, and is widely recognized as the most comprehensive and reliable database for bibliometric analysis [7].

In this study, a literature search was conducted on the Web of Science Core Collection (WoSCC) database (http://www.webofscience.com/wos/woscc/basic-research), within the Science Citation Index Expanded (SCI-Expanded), to identify relevant publications. The search was performed on October 20, 2025, and encompassed the period from January 1, 2005, to December 31, 2024.

Following the acquisition of relevant keywords and their supplementation with Medical Subject Headings (Mesh)subject headings from Pubmed, and based on the other bibliometric search strategy, all topic terms were searched within the article titles (TI), author keywords (AK), and abstracts (AB) [9, 10], and the detailed search query is built and provided in the Appendix.

**Fig. 1:**
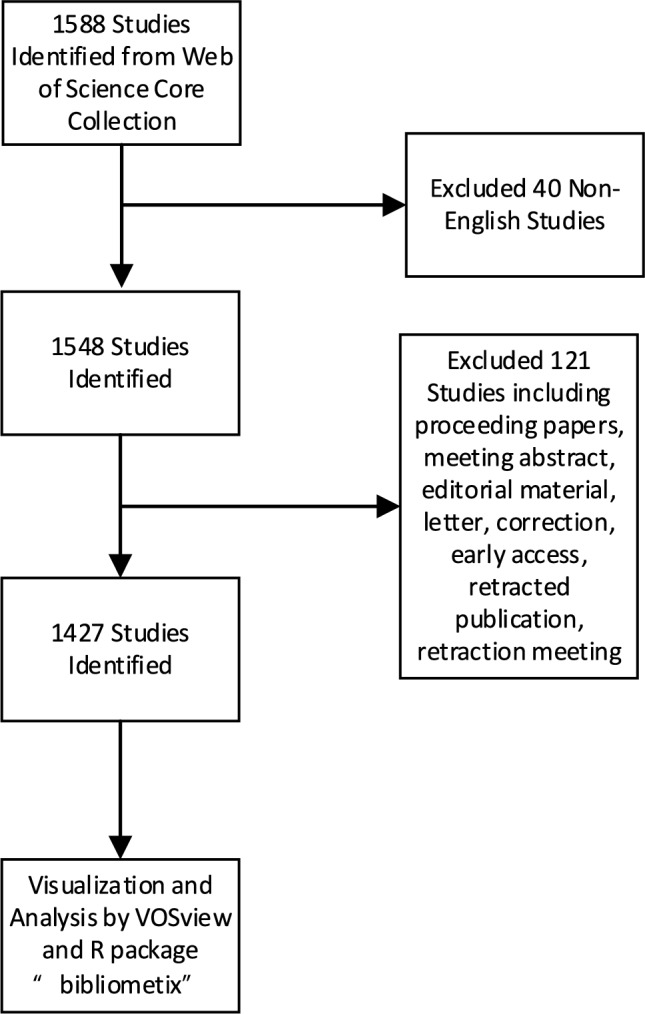
Publications screening flowchart

The search strategy was developed using a stepwise approach. First, to capture all minimally invasive esophagectomy techniques, we combined MeSH terms ('Esophagectomy' and 'Minimally Invasive Surgical Procedures') with supplementary free-text terms not indexed in MeSH but commonly used in the literature, including 'inflatable mediastinoscopy', 'inflatable video-assisted mediastinoscopic transhiatal', and 'robot-assisted'. Second, a separate group was formed using established procedural terminology: 'Thoracoscopic Laparoscopic Esophagectomy', 'Thoracoscopic Esophagectomy', and 'Laparoscopic Esophagectomy'. These two groups were then combined to create a comprehensive set encompassing virtually all minimally invasive esophagectomy approaches. Finally, this set was intersected with the MeSH term 'Cancer of Esophagus' to restrict results to studies focused on esophageal cancer.The initial search yielded 1588 records. To refine the results, the search was limited to publications in the English language. Furthermore, several document types were excluded. Subsequently, only “article” and “review” document types were retained, resulting in 1427 studies for subsequent bibliometric analysis (Fig. 1).

### Data analysis

In this study, bibliometric analysis was performed mainly using three specialized tools: the Biblioshiny web interface (version 5.0) from the R package bibliometrix (R version 4.5.1) [11], VOSviewer (version 1.6.20) [12] and Citespace (version 6.4.R1) [13].

The bibliometrix R package provides a comprehensive suite of functions for quantitative bibliometric research [7, 8]. In this study, Biblioshiny was utilized to (1) calculate and visualize the collaborative networks among different countries/regioins; (2) identify and analyze the most relative journals based on H-index and Bradfordlaw; (3) analyze the author’s keywords, revealing the co-occurrence network, trend topic and thematic map.

VOSviewer is a software tool specifically designed for constructing and visualizing bibliometric networks based on co-occurrence data [14, 15]. It was employed in this study to (1) create a co-authorship network, mapping the collaborative relationships of countries/regions, authors and institutions, and (2) construct a co-cited network of references. In the resulting network maps, the size of the nodes represents the importance or frequency of the item (e.g., an author or a reference), the thickness of line between nodes indicates the strength of their relationship, and the colour coding is used to cluster related items.

Citespace is another bibliometric analysis software developed by Professor Chen C [8, 16].In this study, it was used to identify cited references with the strongest citation burst during the period.

The data retrieved from WoSCC was exported in a plain text format containing full records and cited references, which served as the input for these analytical tools.

The Microsoft Office Excel 365 was used to visualize the publication output trend based on the data from WoSCC.

## Result

### Publication summary

**Fig. 2:**
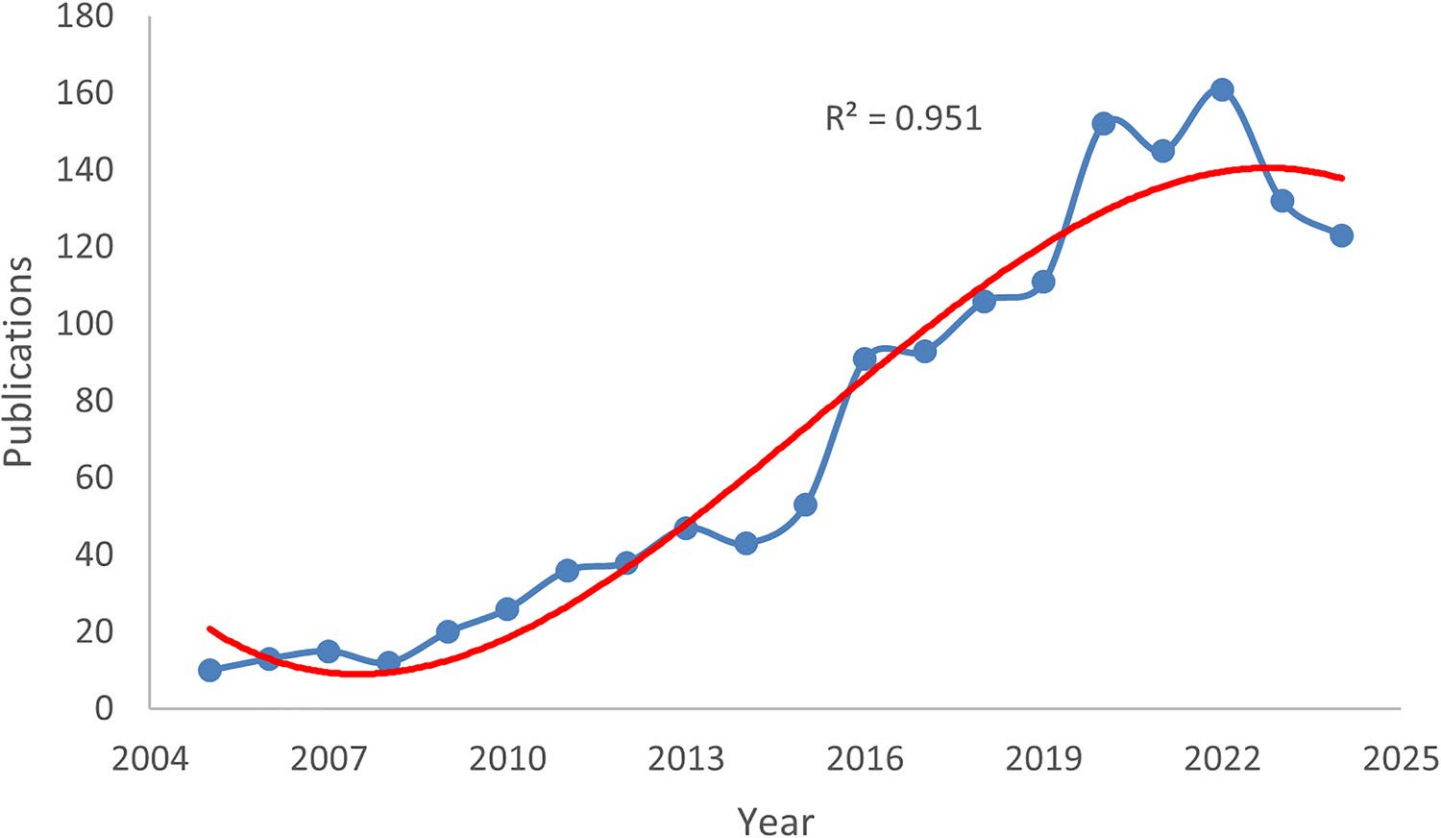
Annual publication volume on MIE and its fitted curve

A total of 1427 English publications were retrieved from the Web of Science (WOS) database, comprising 1270 research articles and 157 review articles. According to the summary data provided by WOS (Web Of Science), these publications have garnered a total of 30,156 citations, with an average of 21.13 citations per article. The H-index for this research field is 74. (Fig. 2) illustrates the annual publication number concerning the application of MIE in esophageal cancer treatment, along with a fitted trend line (*R*^2^ = 0.951). The primary trend indicates an initial increase in publications, followed by a plateau at high levels after 2020, peaking at 161 publications in 2022. The cumulative number of publications has shown continuous growth. Based on this analysis, we believe that MIE represents one of the focal points in the field of esophageal cancer treatment, warranting further investigation into its future directions and developmental drivers.

### Analysis of countries/regions

**Fig. 3:**
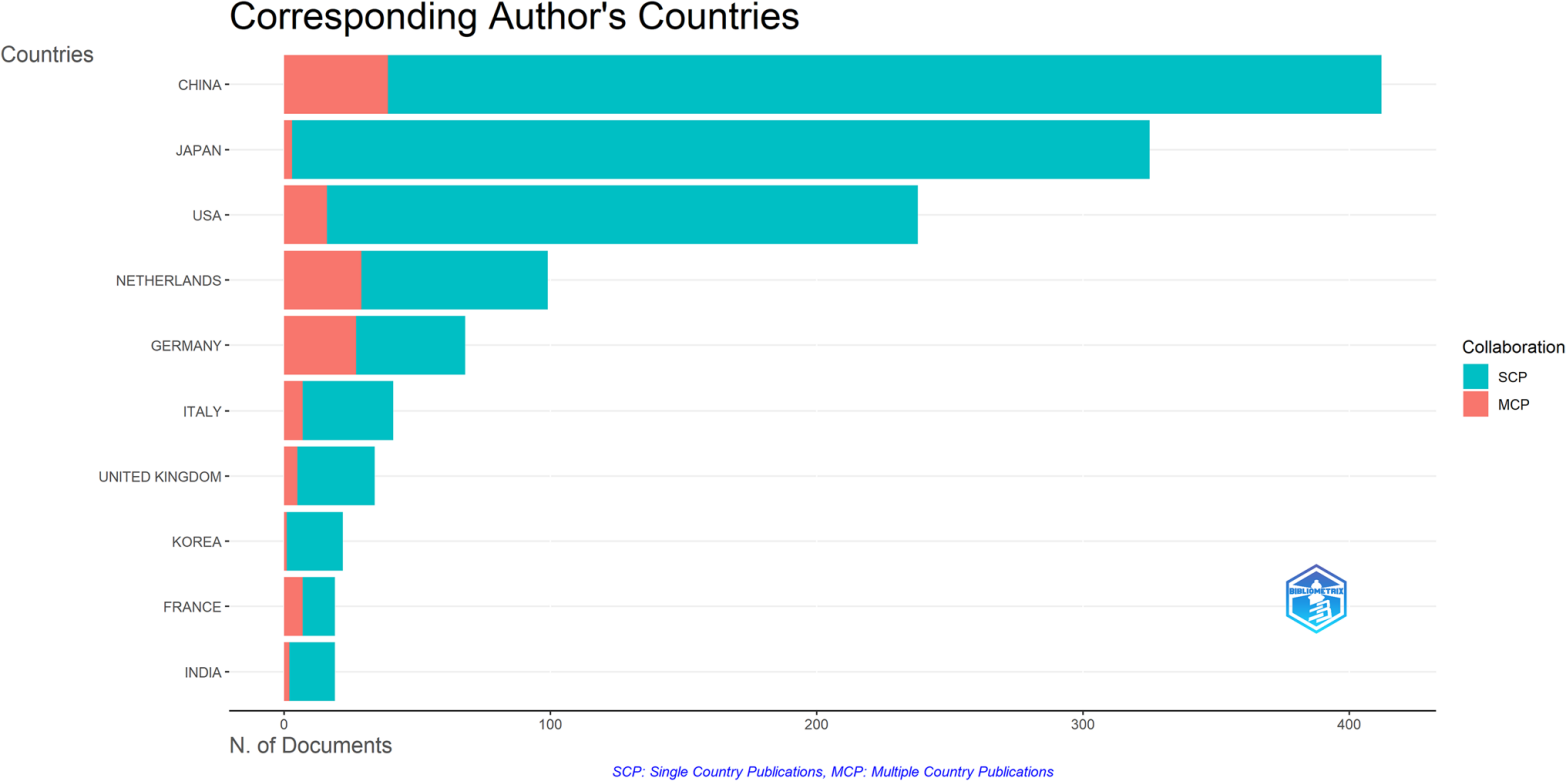
The top ten productive countries/regions based on corresponding author’s countries/regions

From 2005 to 2024, a total of 36 countries/regions contributed to publications in this field (Fig. 3) shows the top ten countries/regions based on the corresponding author's affiliation, also indicating the proportion of international collaborative publications. In the bar chart, the length of each bar represents the total number of publications. Red segments denote single-country publications (SCP), while green segments represent multiple-country publications (MCP), reflecting the extent of international collaboration.China led in output with 412 publications, followed by Japan (325), the USA (238), and the Netherlands (99). Most countries published independently.

**Fig. 4:**
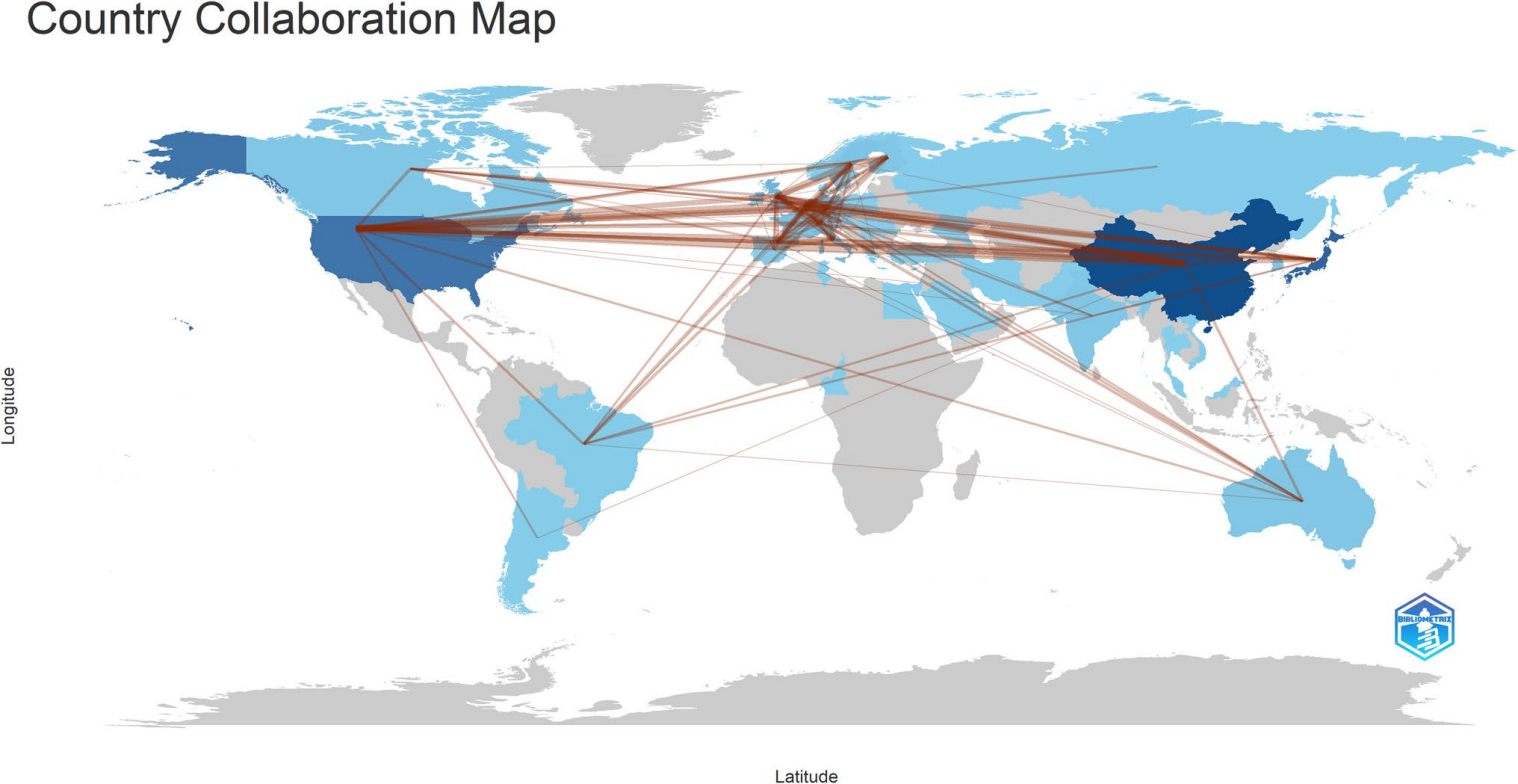
International collaboration patterns visualized on the world map

**Fig. 5:**
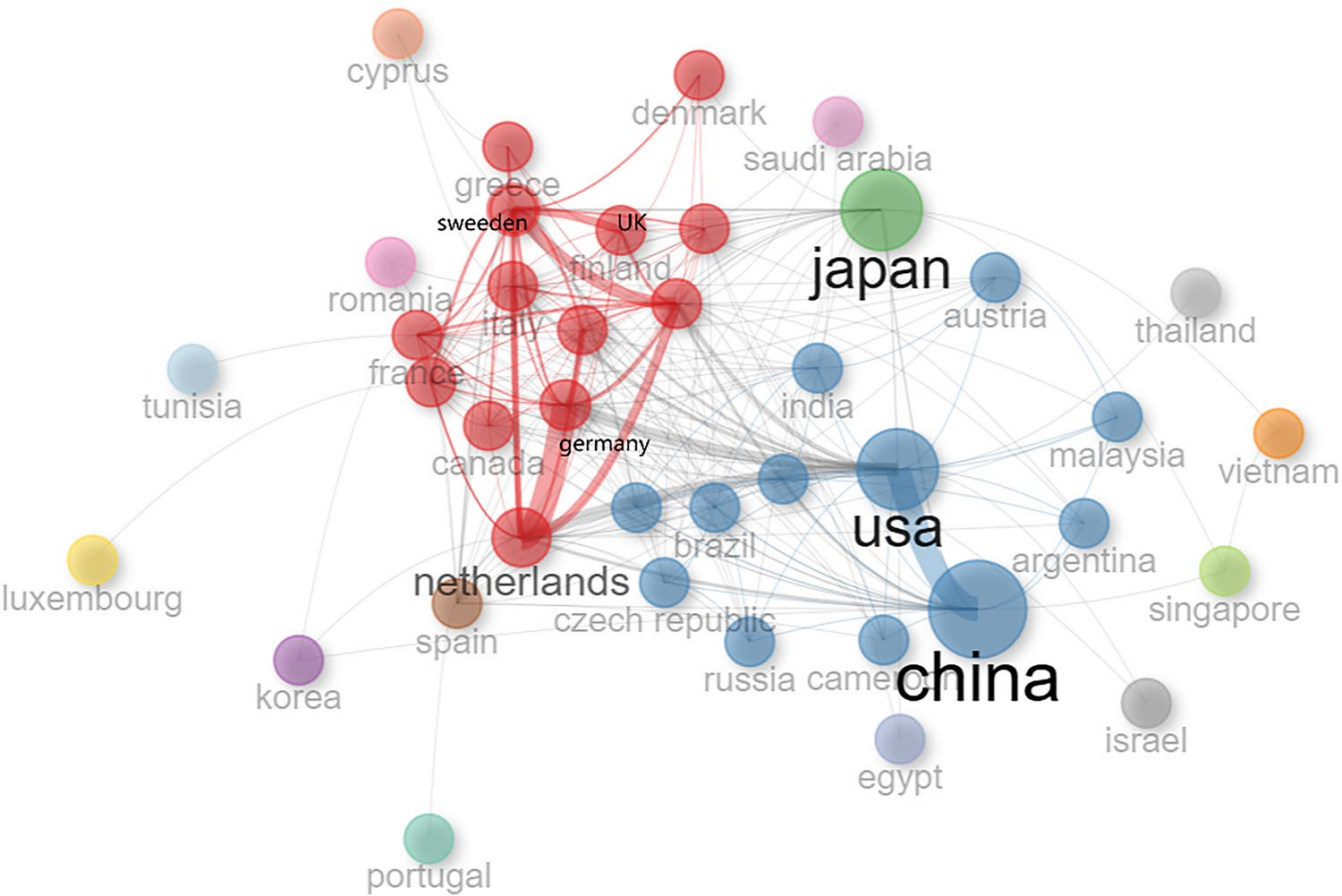
International collaboration patterns by clusters

International collaboration patterns were visualized using a world map (Fig. 4) and a cluster network (Fig. 5). In these visualizations, node size represents the volume of publications, line thickness indicates the relative frequency/intensity of collaboration, and nodes of the same color belong to the same collaborative cluster. Three primary collaborative clusters were identified: a major cluster centered on China and the USA; a European-centric cluster involving the Netherlands, Germany, Finland, and Sweden; and a relatively independent network around Japan. Notably, despite its high publication output (ranking second overall), Japan exhibited limited international collaboration, resulting in its identification as a relatively independent cluster in the network analysis. This finding aligns with the country-wise publication data presented in Fig. 3, which shows Japan's high proportion of single-country publications (SCP).

The top ten countries/regions by total citations are listed in Table 1. The USA accumulated the highest total citations (6457), followed by Japan (6069), China (5466), and the Netherlands (4339), consistent with the top four publishing nations. Japan ranked second in both publication count and total citations.

**Table 1:** The top 10 countries/regions by total citations

Country	Total citations	Average article citations
USA	6457	27.1
Japan	6069	18.7
China	5466	13.3
Netherlands	4339	43.8
United Kingdom	1259	37.0
Germany	1248	18.4
France	1058	55.7
Australia	1018	67.9
Italy	546	13.3
India	493	25.9

### Analysis of institutions

**Fig. 6:**
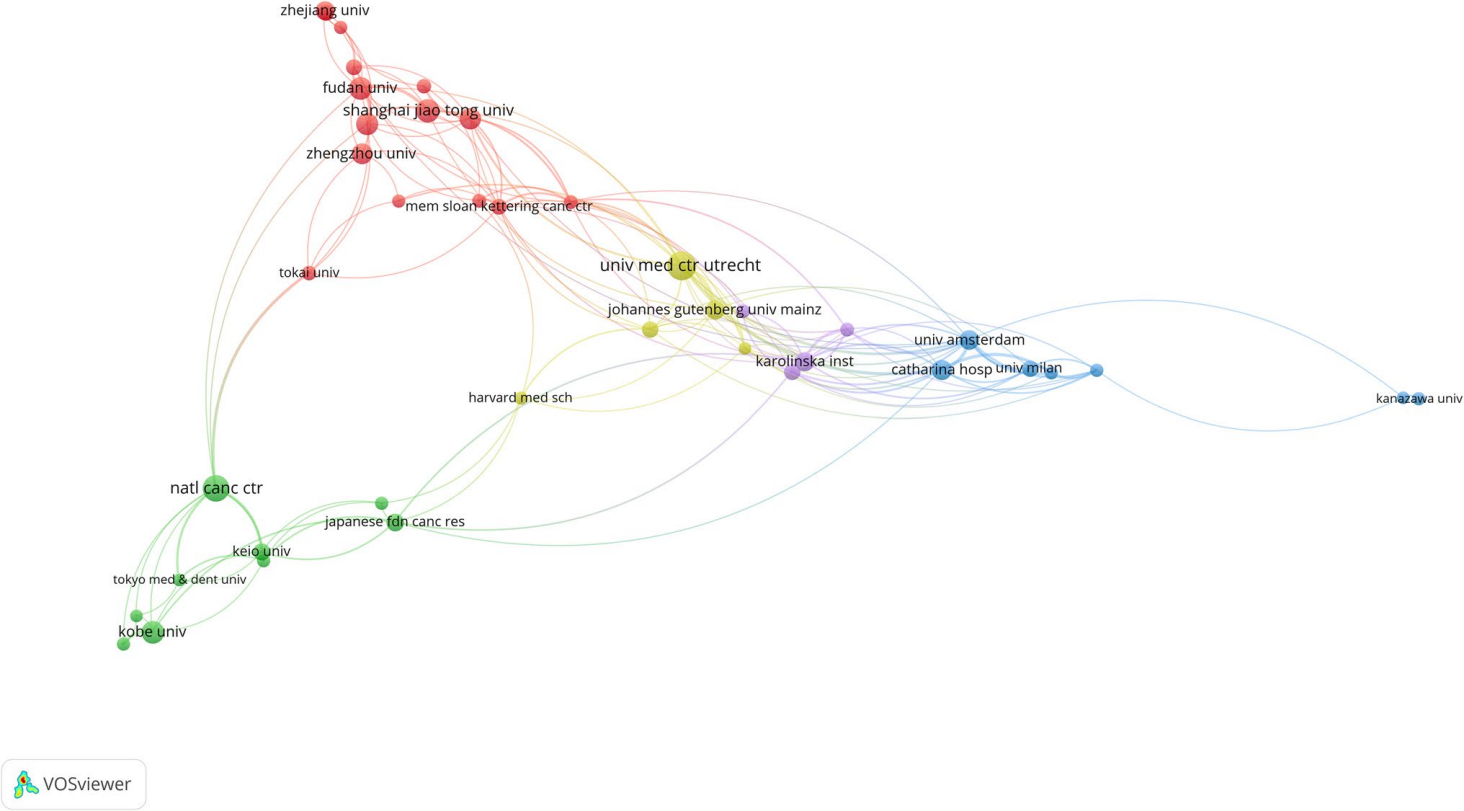
The network visualization of institutional collaborations

A total of 1251 institutions contributed to the publications in this field. The top three most productive institutions were The University Medical Center Utrecht (Netherlands), the National Cancer Center (Japan), and Shanghai Jiao Tong University (China) (Table 2). The network visualization of institutional collaborations (Fig. 6) reveals that partnerships predominantly form clusters confined within the same countries/regions. In this visualization, node size represents publication volume, and institutions sharing the same color belong to the same collaborative cluster. For example, Shanghai Jiao Tong University and Fudan University (China) form the red cluster; the National Cancer Center Japan and Keio University (Japan) form the green cluster; and the University Medical Center Utrecht (Netherlands) collaborates closely with European partners such as Johannes Gutenberg University Mainz (Germany), forming the yellow cluster. Karolinska Institutet (Sweden) is part of the purple cluster, while Ca' Granda Hosp, University of Milan (Italy) belongs to the blue cluster, highlighting their notable contributions from the perspective of collaborating institutions. Notably, the yellow, purple, and blue clusters exhibit relatively dense connections, reflecting strong collaborative ties within Europe.

**Table 2:** The top 10 productive institutions

Institution	Country/Region	Articles published	Cited	Average article citations
The University Medical Center Utrecht	Netherlands	55	2276	41.4
National Cancer Center Japan	Japan	45	870	19.3
Shanghai Jiao Tong University	China	35	1088	31.1
Fudan University	China	34	1264	37.2
Kobe University	Japan	34	613	18.0
Fujian Medical University	China	31	469	15.1
University of Pittsburgh	USA	30	858	28.6
Zhengzhou University	China	28	299	10.7
Catharina Hospital	Netherlands	27	884	32.7
Sichuan University	China	27	364	13.5

### Analysis of journals

**Fig. 7:**
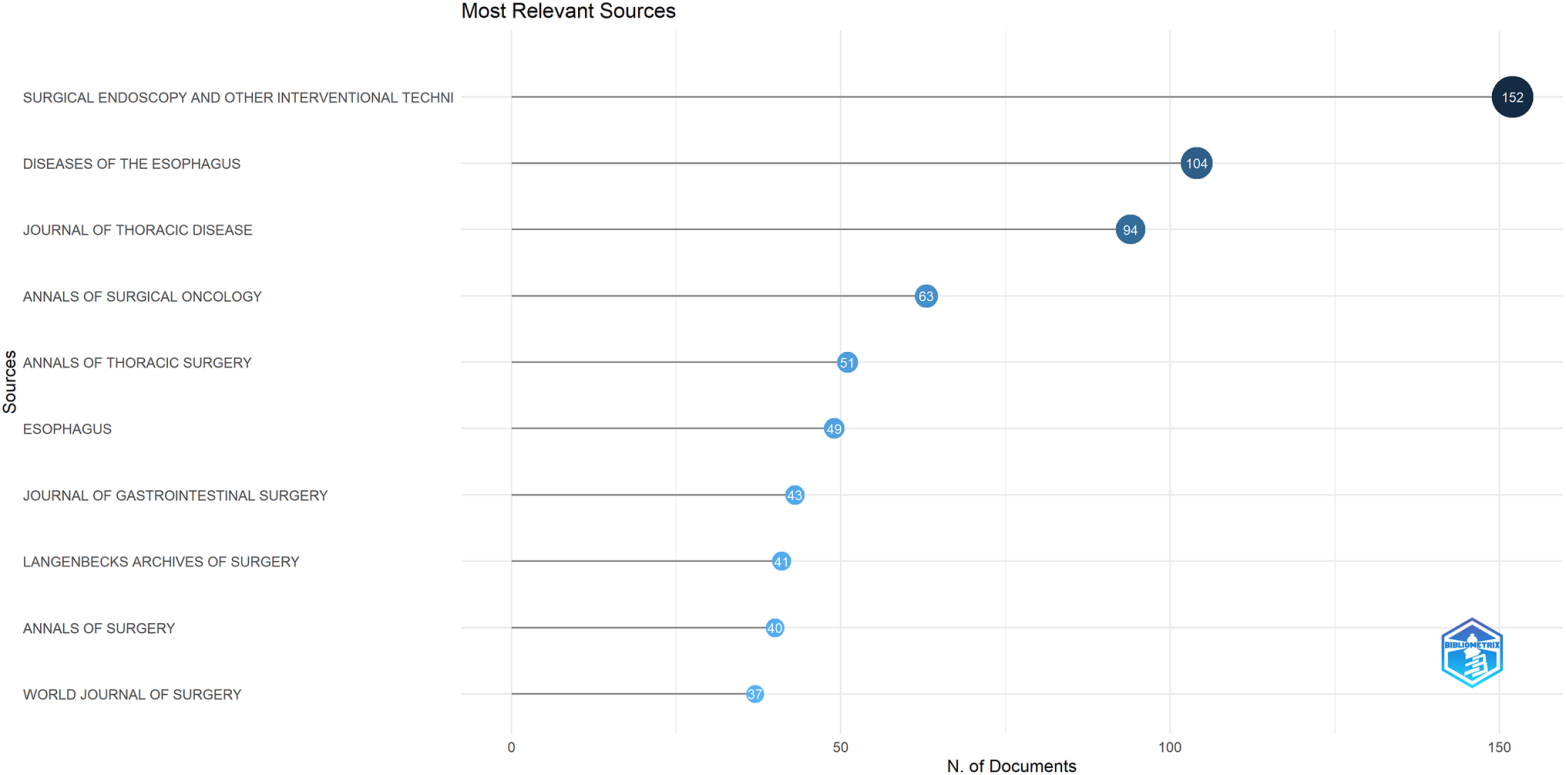
The top ten productive journals

**Fig. 8:**
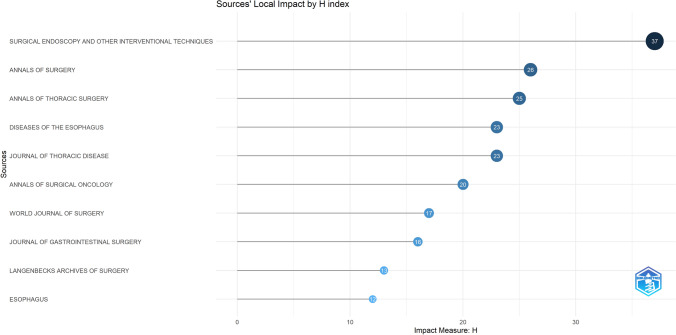
The top ten journals ranked by H-index

**Fig. 9:**
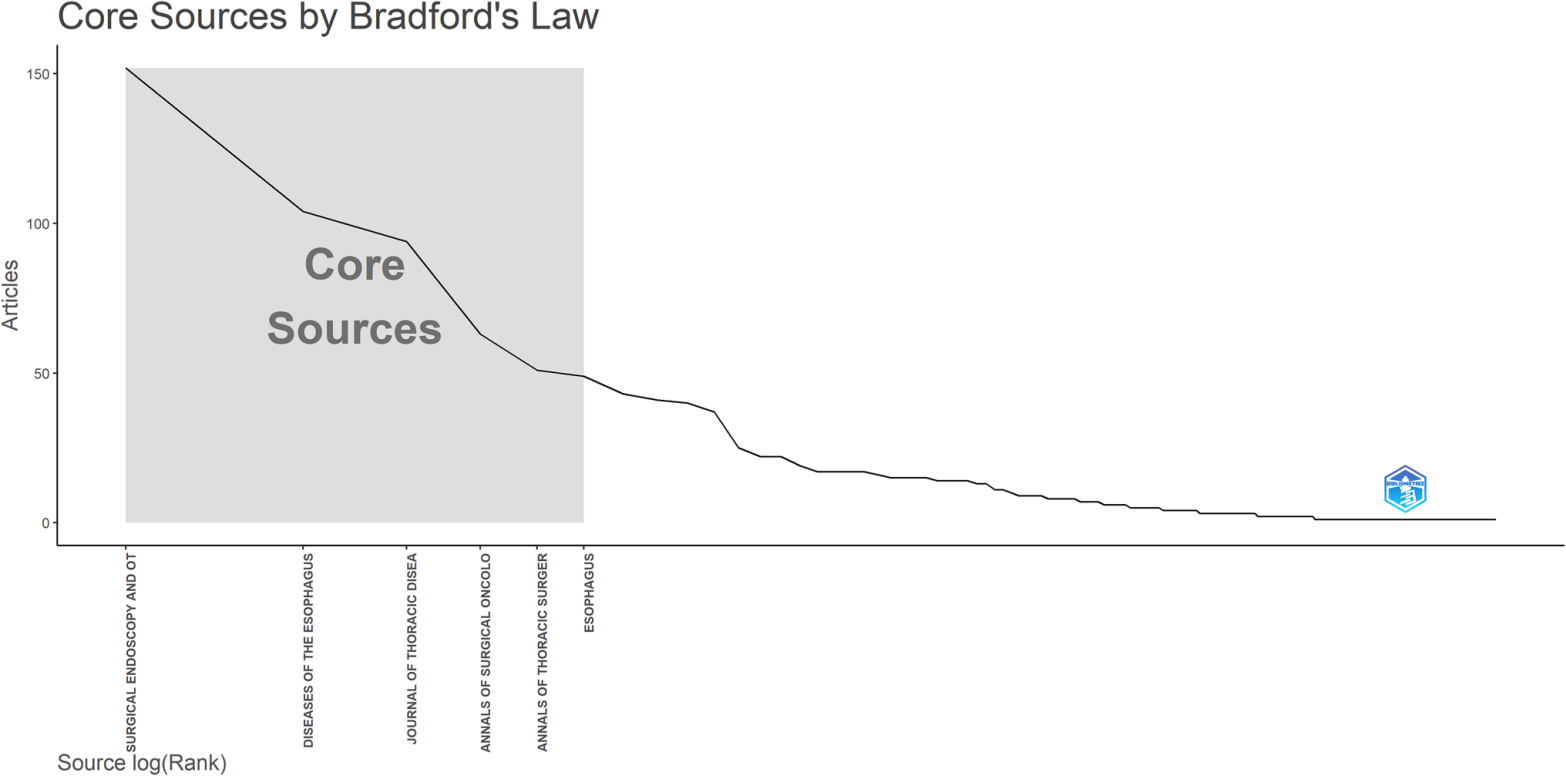
The journals ranked according to Bradford's Law

A total of 213 journals published relevant studies in this field. The top ten journals by publication volume are shown with their values in Fig. 7, with the top three being *Surgical Endoscopy and Other Interventional Techniques*, *Diseases of the Esophagus*, and *Journal of Thoracic Disease*. The top ten journals ranked by H-index [17] and their H-index values are presented in Fig. 8. According to Bradford's Law, the core journals, visualized in Fig. 9 and detailed in the Table 3, consist of the top six journals by publication count. There is considerable overlap among the leading journals across these three metrics.

**Table 3:** The core journals by Bradford's Law

Journal	H-index	IF (2020)	IF (2021)	IF (2022)	IF (2023)	IF (2024)
Surgical Endoscopy and Other Interventional Techniques	37	4.6	3.5	3.1	2.4	2.7
Diseases of the Esophagus	23	3.4	2.8	2.6	2.4	2.3
Journal of Thoracic Disease	23	2.9	3.0	2.5	2.1	1.9
Annals of Surgical Oncology	23	5.3	4.3	3.7	3.4	3.5
Annals of Thoracic Surgery	25	4.3	5.1	4.6	3.7	3.9
Esophagus	12	4.2	3.7	2.4	2.2	3.8

*IF* impact factor

### Analysis of references

**Fig. 10:**
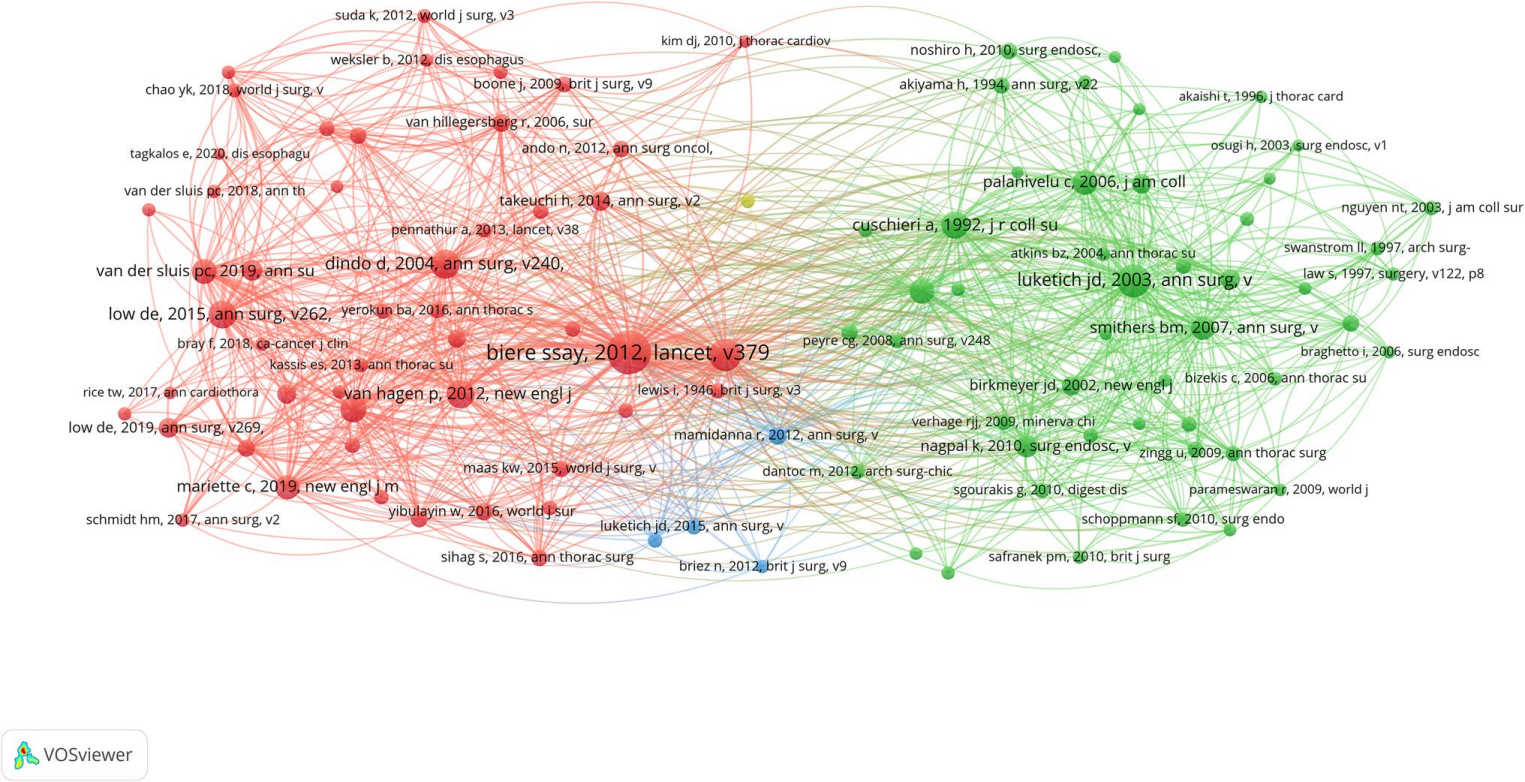
The co-cited references network

**Fig. 11:**
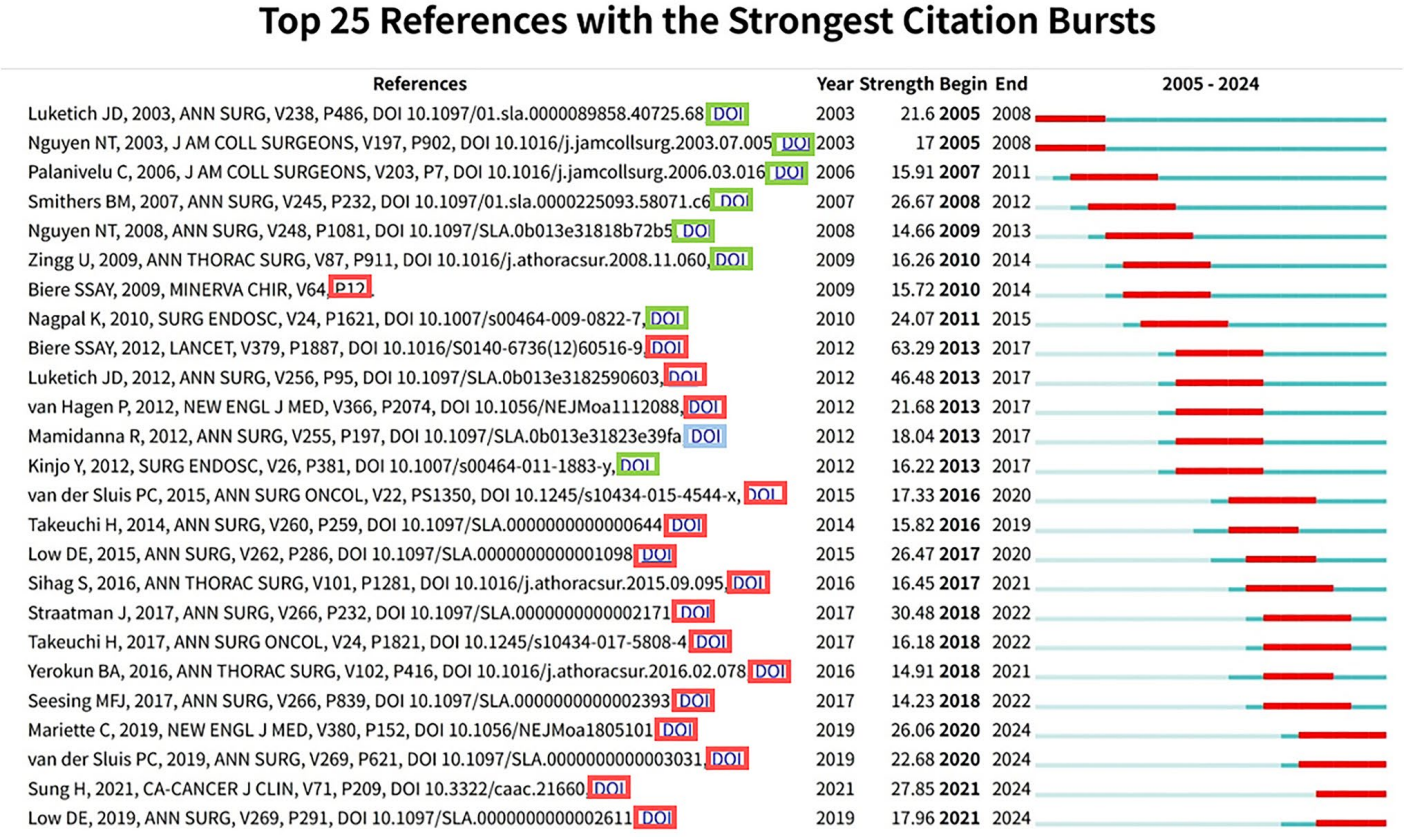
Top 25 references with the strongest citation bursts

Within this research field, a total of 15,157 references have been cited. The ten most frequently co-cited references, detailing their respective journals, authors, and country/region of the author, are presented in the (Table 4). Applying a minimum co-citation frequency threshold of 40 identified 102 references, the network of which is visualized in Fig. 10. Additionally, 25 references exhibiting significant citation bursts are identified and illustrated in Fig. 11.

**Table 4:** The top 10 co-cited references

DOI	Journal	Country/Region	Corresponding author	Publication Year	Citations
10.1016/s0140-6736(12)60,516–9	Lancet	Netherlands	Biere, Surya S. A. Y	2012	496
10.1097/01.sla.0000089858.40725.68	Annals of Surgery	USA	Luketich, James D	2003	277
10.1097/sla.0b013e3182590603	Annals of Surgery	USA	Luketich, James D	2012	265
10.17116/hirurgia2018090162 10.1097/01.sla.0000133083.54934.ae	Annals of Surgery	Switzerland	Dindo, D	2004	224
10.1056/nejmoa1112088	New England Journal of Medicine	Netherlands	van Hagen, P	2012	202
10.1097/sla.0000000000001098	Annals of Surgery	USA	Low, Donald E	2015	199
j r coll surg edinb, v37, p7	Surgical Oncology-Oxford	Scotland	Cuschieri, A	1993	189
10.1097/sla.0000000000002171	Annals of Surgery	Netherlands	Straatman, Jennifer	2017	173
10.1097/sla.0000000000003031	Annals of Surgery	Netherlands	van der Sluis, Pieter C	2019	161
10.1056/nejmoa022343	New England Journal of Medicine	Netherlands	Hulscher, JBF	2002	158

*DOI* digital object identifier

As shown in Figs. 10 and 11 and Table 4, the co-cited references can be broadly categorized into two main clusters, distinguished by green and red coloring (reflected in the row backgrounds of Table 4, the node colors in Fig. 10, and the bars highlighting DOIs in Fig. 11). The green cluster predominantly comprises earlier studies focusing on the initial application of minimally invasive esophagectomy (MIE), primarily summarizing preliminary findings on surgical safety and efficacy. In contrast, the red cluster represents more recent research, marked notably by the 2012 randomized controlled trial (RCT) comparing open and minimally invasive surgery conducted by Biere, Surya S. A. Y. (Netherlands) and the study on neoadjuvant therapy for esophageal cancer by P. van Hagen (Netherlands). These studies, drawing on larger surgical case series, longer follow-up periods, and RCT evidence, have validated the effectiveness of MIE and neoadjuvant therapy. Furthermore, definitions of postoperative complications have become increasingly standardized within this cluster, facilitating more reliable comparisons of outcomes across different surgical approaches.

### Analysis of authors

**Fig. 12:**
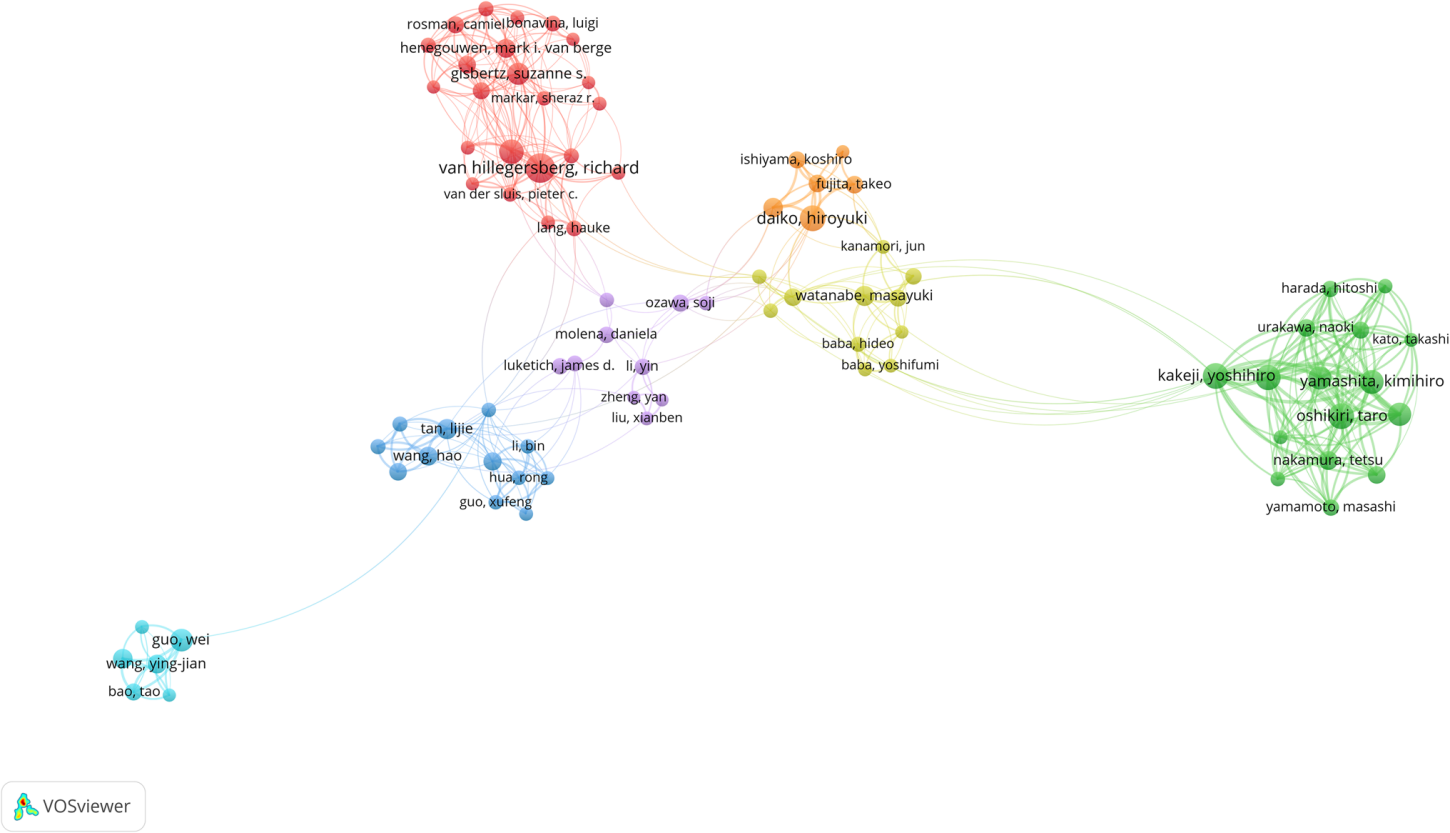
The network of co-occurrence of the authors

This analysis encompassed 6125 authors (Table 5) presents the top 10 authors ranked by number of publications. The leading contributors were van Hillegersberg, Richard (Netherlands), Daiko,Hiroyuki (Japan), and Kakeji, Yoshihiro (Japan), focusing on robot-assisted minimally invasive esophagectomy, and developments in neoadjuvant therapy and esophagectomy in Japan, respectively. A co-occurrence analysis of the top 100 authors (minimum publications = 9) is illustrated in Fig. 12, indicating that research collaboration is predominantly intra-national, exemplified by the distinct cluster for Japan (in green). The top 10 most co-cited authors are listed in (Table 6), led by Luketich, James D. (USA), Biere, Surya S. A. Y. (Netherlands), and van der Sluis, Pieter C. (Netherlands). Their key research areas comprise comprehensive reviews of esophageal cancer, and randomized controlled trials evaluating minimally invasive esophagectomy versus open surgery, as well as robot-assisted minimally invasive esophagectomy (RAMIE) versus open surgery, respectively.

**Table 5:** The top 10 productive authors

Name	Country/Region	Institution	Article	Total Citatioins	DOI (highly cited article by the author)
van Hillegersberg, Richard	Netherlands	Univ Med Ctr Utrecht	44	1858	10.1097/SLA.0000000000003031
Daiko, Hiroyuki	Japan	Natl Canc Ctr Hosp East	34	457	10.1093/jjco/hyt061
Kakeji, Yoshihiro	Japan	Kyushu Univ	33	538	10.1016/j.surg.2007.12.007
Oshikiri, Taro	Japan	Kobe Univ	33	426	10.1007/s10388-020–00782-1
Kanaji, Shingo	Japan	Kobe Univ	32	397	10.1007/s00464-020–07455-1
Ruurda, Jelle P	Netherlands	Univ Utrecht	31	1581	10.1158/1078–0432.CCR-20–4443
Yamashita, Kimihiro	Japan	Kobe Univ	29	385	10.1007/s00464-020–07455-1
Hasegawa, Hiroshi	Japan	Hyogo Canc Ctr	28	364	10.1007/s00464-014–3919-6
Matsuda, Takeru	Japan	Kobe Univ	27	346	10.21873/anticanres.13631
Guo, Wei	China	Fudan Univ	26	351	10.1007/s00464-015–4692-x

*DOI* digital object identifier

**Table 6:** The top 10 most co-cited authors

Author	Country/Region	Institution	Total citations	DOI (highly cited article by the author)
Luketich, James D	USA	Univ Pittsburgh	701	10.1016/S0140-6736(12)60,643–6
Biere, Surya S. A. Y	Netherlands	Vrije Univ Amsterdam Med Ctr	631	10.1016/S0140-6736(12)60,516–9
van der Sluis, Pieter C	Netherlands	Netherlands	408	10.1186/1745–6215-13–230
Low, Donald E	USA	Virginia Mason Med Ctr	373	10.1097/SLA.0000000000001098
Mariette, Christophe	France	Univ Hosp Claude Huriez	332	10.1200/JCO.2005.04.7118
Nguyen, NT	USA	Univ Calif Davis	288	10.1001/archsurg.135.8.920
Hulscher, JBF	Netherlands	Univ Amsterdam	258	10.1056/NEJMoa022343
CUSCHIERI, A	SCOTLAND	University of Dundee	256	10.1016/0960–7404(93)90,052-Z
Takeuchi, Hiroya	Japan	Japanese Soc Gastroenterol Surg	241	10.1097/SLA.0000000000000644
Rice, Thomas W	USA	Cleveland Clin	234	10.21037/acs.2017.03.14

*DOI* digital object identifier

### Analysis of keywords

**Fig. 13:**
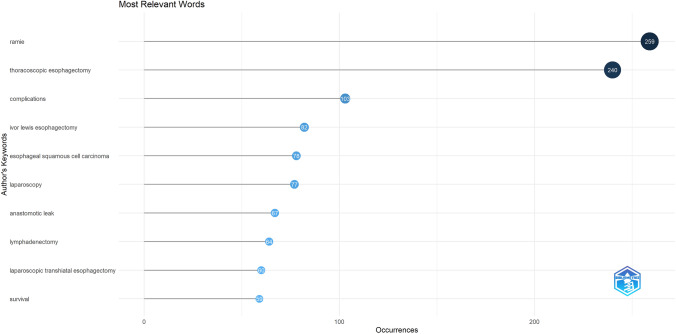
The top 10 most relevant words

**Fig. 14:**
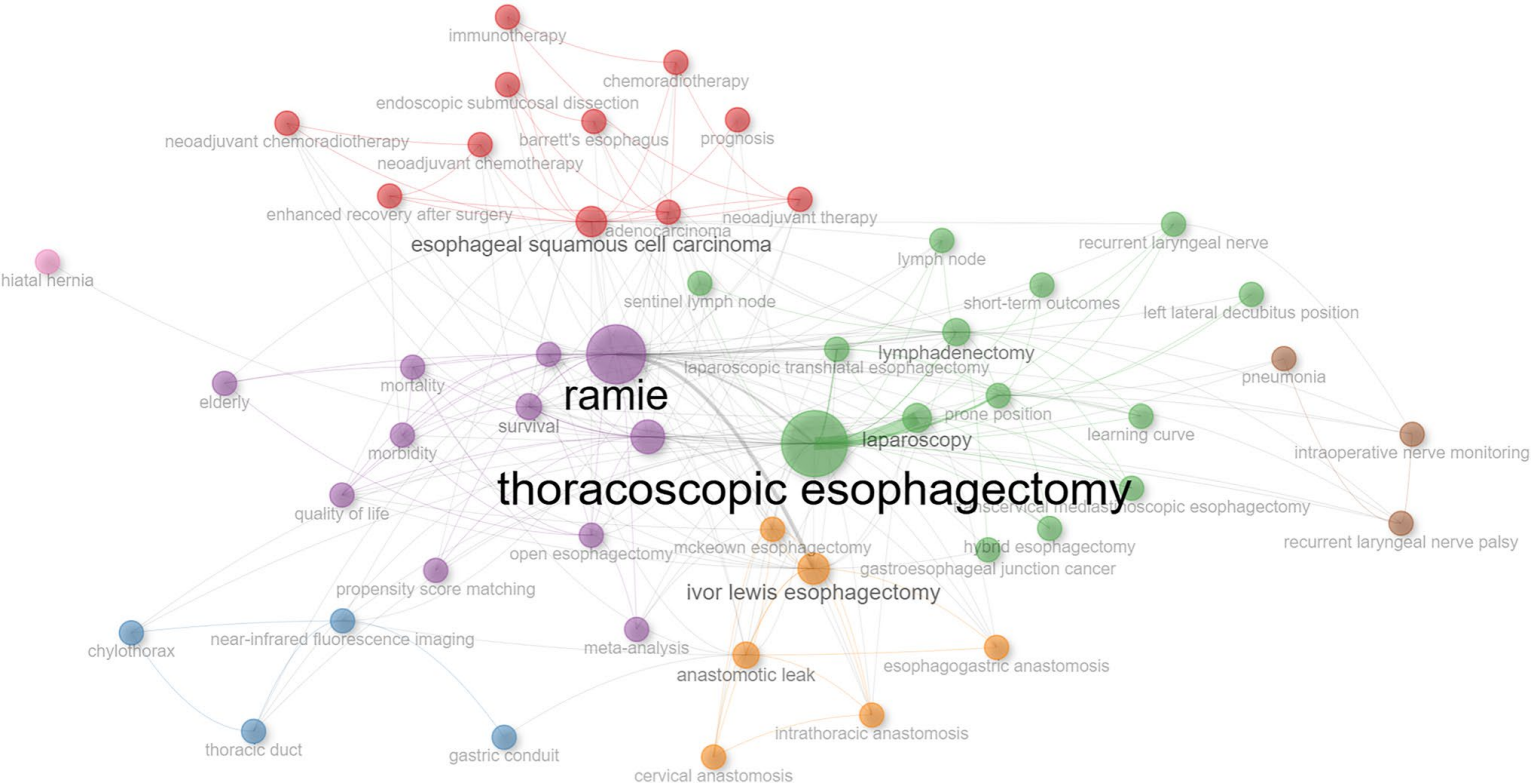
The network of keywords co-occurrence

The analysis identified 1013 author’s keywords. The top ten keywords are presented in Fig. 13, led by RAMIE (Robot-assisted minimally invasive esophagectomy), thoracoscopic esophagectomy, and complications. Keyword co-occurrence analysis clustered the terms into four primary categories, illustrated in Fig. 14. Cluster 1 (purple) is associated with RAMIE, mainly involving evaluations of this surgical approach. Cluster 2 (green) revolves around thoracoscopic-laparoscopic esophagectomy, encompassing studies on aspects of this foundational minimally invasive procedure, such as lymphadenectomy and patient positioning. Cluster 3 (red) focuses on neoadjuvant therapy for esophageal squamous cell carcinoma and strategies to improve surgical outcomes and survival. A smaller, fourth cluster (orange) is dedicated to research on the Ivor Lewis esophagectomy technique. Additional minor clusters addressed topics like the use of near-infrared fluorescence imaging and recurrent laryngeal nerve preservation.

**Fig. 15:**
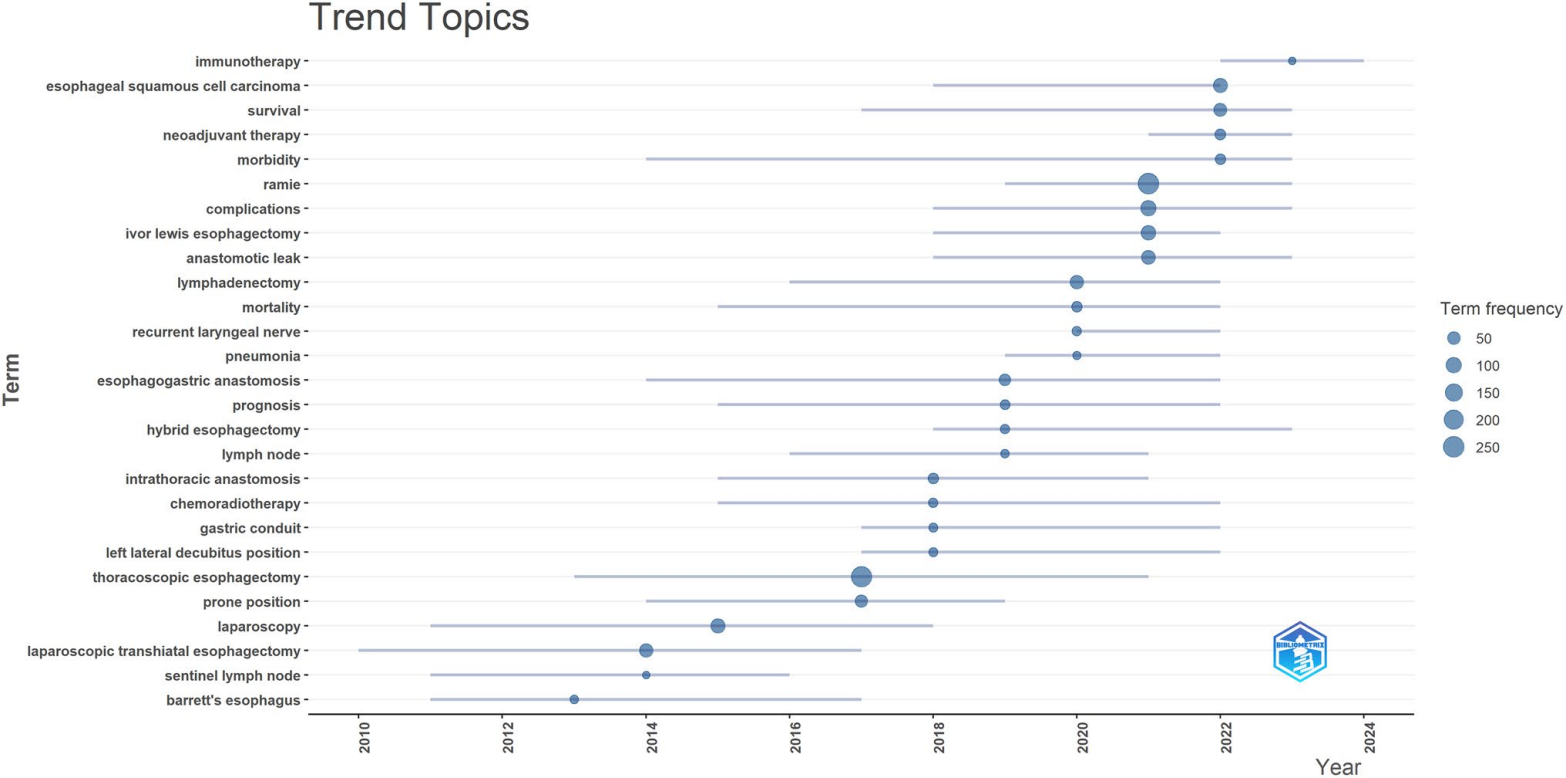
The trend of research topic

The trend of research topics (Fig. 15), generated with a minimum word frequency of 10 and 4 words per year. This analysis, based on the underlying algorithm of the package, identifies research hotspots by tracking keywords that exhibit significantly increased frequencies within specific time periods [11]. The results indicate an evolution from an initial focus on surgical techniques and complications towards neoadjuvant and adjuvant therapies. Notably, there is a rising interest in combining immunotherapy with surgery to improve prognosis.

**Fig. 16:**
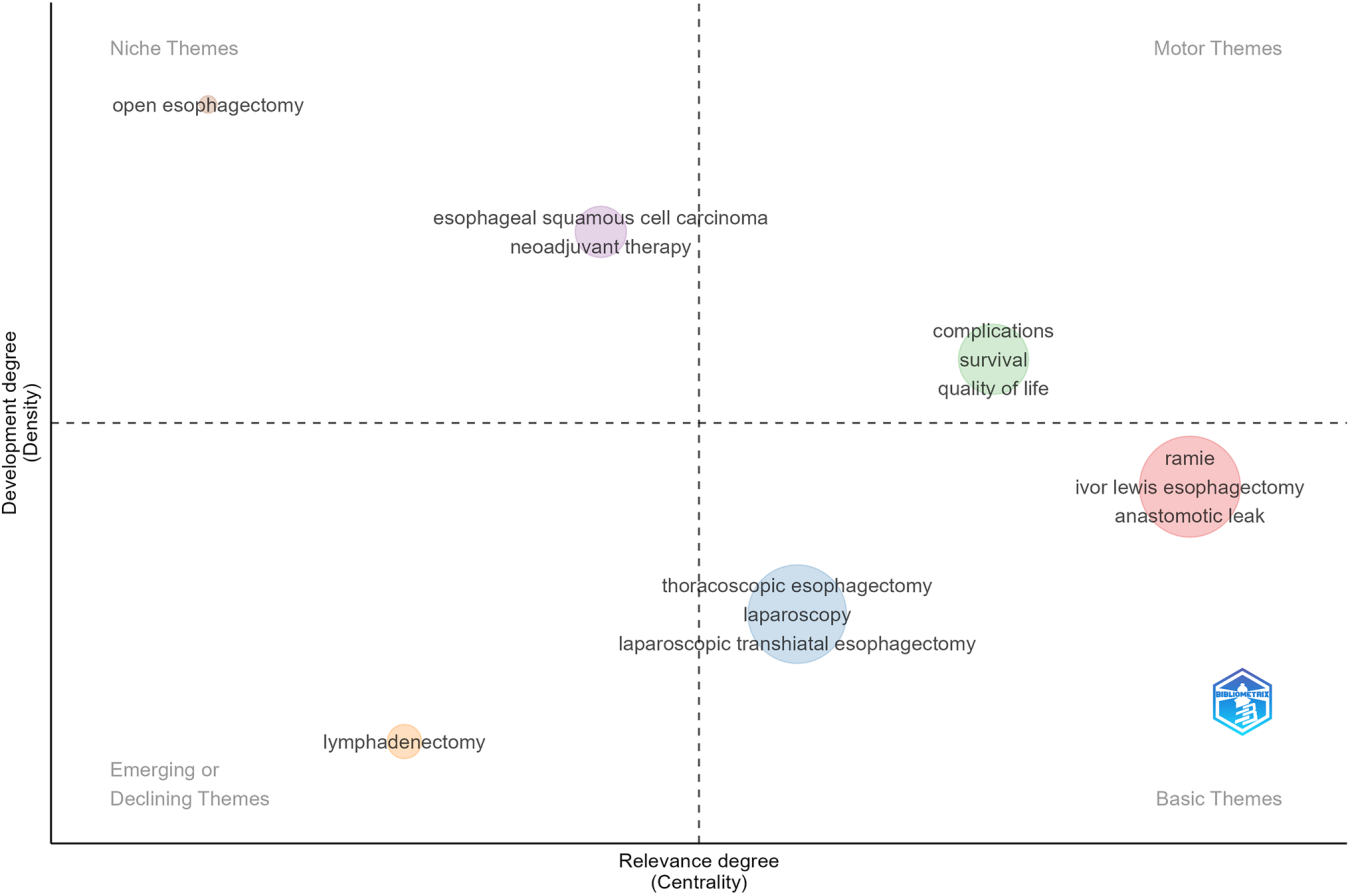
The thematic map

The thematic map (Fig. 16), created with 250 words and a minimum cluster frequency of 20, delineates the research landscape. The horizontal axis (centrality) represents the degree of relevance of different thematic clusters, reflecting the strength of their external connections to other themes. The vertical axis (density) represents the internal coherence among keywords within a cluster, indicating the developmental level of each theme.

Based on their positioning in the four quadrants, the identified themes can be categorized as follows: Evaluation metrics for minimally invasive techniques constitute the motor themes, these are transversal topics with high centrality but relatively lower density, meaning they are foundational to the field and connected to multiple other themes, yet their internal development is more diffuse. The basic themes comprise the various minimally invasive esophagectomy procedures themselves, these are transversal topics with high centrality but relatively lower density, meaning they are foundational to the field and connected to multiple other themes, yet their internal development is more diffuse.The niche themes include two relatively specialized and well-developed areas: one concerning research on esophageal squamous cell carcinoma and neoadjuvant therapy, which shows potential for interdisciplinary linkage, and another on open esophagectomy, which is primarily studied in comparison to minimally invasive approaches, representing a relatively independent and mature area of investigation. Lymphadenectomy appears as an emerging or declining theme, suggesting that future research direction in this domain is influenced by multiple factors and remains uncertain.

## Discussion

This study provides a bibliometric analysis and knowledge mapping of the minimally invasive esophagectomy (MIE) for esophageal cancer over the last twenty years, offering a novel research not previously available.

Publication output peaked in 2022 and has since plateaued, followed by a modest decline. This pattern of stabilization likely reflects the maturation of combined thoracoscopic-laparoscopic esophagectomy as a standard approach [18, 19], where further refinement is now tied to advancements in surgical technology, such as robotic systems [20]. Concurrently, the variety of surgical techniques (e.g., laparoscopic transhiatal with/without transcervical approaches) and their integration with diverse neoadjuvant/adjuvant regimens create multiple treatment pathways [21, 22].

Evaluating these multimodal strategies requires large-scale trials and mature survival data. The time needed to gather such longitudinal data naturally leads to a temporary slowdown in publications. Future research growth will likely depend on breakthroughs in surgical technology—such as the broader adoption of robotic systems (discussed later)—or on conclusive results from maturing datasets. However, these advances require not only innovation but also cost reductions, creating a temporal lag that delays a resurgence in output. Thus, the recent decline may represent a period of consolidation, during which the field awaits transformative evidence or technological breakthroughs to spark renewed research activity. Bibliometric analysis identifies China, Japan, the USA, and the Netherlands as the most prolific contributors. When citation impact is considered, the Netherlands stands out for producing high-quality research despite a smaller volume, an advantage facilitated by comprehensive national cancer registries enabling early randomized controlled trials (RCTs) [19]. International collaboration patterns are noteworthy: Japan, while second in output and citations, shows limited co-authorship with other nations. In fact, based on our data, Japan's multiple-country publication (MCP) ratio is only 0.9%, compared to 9.4% for China, 6.7% for the USA, and 30% for the Netherlands, as shown in Fig. 3. This may be attributed to epidemiological differences (squamous cell carcinoma dominance in East Asia [22, 23] vs. adenocarcinoma in the West [24]) leading to distinct research priorities, such as Japan's focus on advanced lymphadenectomy techniques [25, 26], and its correspondingly more detailed lymph node classification system and slightly different N-staging criteria based on the Japan Esophageal Society (JES) system versus the Union for International Cancer Control/American Joint Committee on Cancer (UICC/AJCC) system [23, 27, 28], as well as divergent neoadjuvant treatment approaches for locally advanced disease [22, 29, 30]. Japan's relative independence in research also stems from its capacity to conduct high-quality clinical studies through established networks such as the Japan Clinical Oncology Group (JCOG).

This pattern of regionally concentrated collaboration contributes to existence of separate regional clinical guidelines (Chinese Society of Clinical Oncology -CSCO, JES, National Comprehensive Cancer Network-NCCN, European Society for Medical Oncology-ESMO) for the esophageal cancer. However, the definitive assessment of surgical and treatment strategies will ultimately rely on large-scale, multi-national RCTs, which are crucial for establishing standardized, patient-beneficial protocols.

Institutional analysis mirrors national trends, with universities and specialized cancer centers being the primary contributors. Citation-per-article metrics highlight two Dutch universities as leaders in research quality. Notably, five of the top ten publishing institutions are based in China, indicating substantial domestic research capacity and the potential for quality enhancement through intensified collaboration. Currently, significant institutional partnerships remain largely intra-national.

Journal analysis reveals that a consistent set of field-specific journals publishes the bulk of MIE research, as indicated by publication counts, H-index [17], and Bradford's law [31]. However, the journals publishing the most frequently co-cited, foundational papers are high-impact, general medical journals like *The Lancet* and *Annals of Surgery* (Table 4). This suggests that while core specialty journals disseminate much of the field's ongoing work, seminal studies that shape future RCTs and major trials often appear in broader, high-impact venues.

Analysis of co-citation networks points to key research directions. Highly co-cited and burst-strength references predominantly involve RCTs or large cohort studies with long-term follow-up, aimed at proving the efficacy of techniques or treatment models [22, 32], and efforts to standardize the measurement of postoperative complications [33, 34] for safety assessment. This underscores that future, high-impact research will likely involve globally coordinated, large-sample RCTs with prolonged follow-up to definitively establish superiority, building upon earlier pilot studies.

Author network analysis shows the densest collaboration within Japan, centered notably at Kobe University, indicating concentrated expertise and strong institutional leadership. While large trials feature prominently among highly cited works, these authors also contribute influential papers on specific technical aspects like recurrent laryngeal nerve lymphadenectomy [25, 26]. The most co-cited authors are primarily from the USA and the Netherlands, contributing landmark RCTs, guidelines, and consensus statements, alongside pioneers like CUSCHIERI, A (Scotland) for right thoracoscopic esophagectomy [35].

Keyword analysis delineates research evolution and hotspots. MIE, particularly the combined thoracoscopic-laparoscopic approach, is now a mature and widely adopted standard (Fig. 14), forming the basis for both Mckeown and Ivor Lewis procedures [18, 19, 36–39]. Robotic-assisted MIE (RAMIE) is a major current focus (Figs. 13, 14). The enhanced visualization, dexterity, and precision of robotic systems present a significant technological advancement, acting as a powerful surgical adjunct with potential to improve outcomes [3, 20, 39–41]. Ongoing innovation, including single-port [42] and force feedback robots, aims for greater minimal invasiveness and precision [43, 44]. A critical remaining question is cost-effectiveness, which must be addressed for broader adoption [45–47]. Thus, future progress depends on both technological refinement and cost reduction.

Alternative, non-transthoracic approaches like laparoscopic transhiatal [21, 40] (with/without transcervical) esophagectomy represent another active research area (Fig. 16), offering potential advantages such as single-position surgery and reduced operative time [48–50].

Subsidiary technical topics—including patient positioning [51–53], lymphadenectomy extent [54–56], recurrent laryngeal nerve preservation [25, 57, 58], and thoracic duct management [59–61]—form defined but less dominant clusters. While important, some consensus exists, albeit with ongoing debate, making them less central to current debate.

A paradigm shift is occurring in the treatment of esophageal squamous cell carcinoma (ESCC) with the integration of immunotherapy [62–64]. Neoadjuvant chemoimmunotherapy shows promising efficacy [65–67], making this a rapidly growing research frontier (Figs. 15, 16) that intersects deeply with medical oncology and molecular biology [68]. Questions regarding overall survival benefit and optimal adjuvant strategies remain under active investigation.

For esophageal adenocarcinoma (EAC), more common in the West, research focuses on refining the minimally invasive Ivor Lewis procedure and transhiatal esophagectomy [69], particularly overcoming the technical challenges of intrathoracic anastomosis [70, 71] (Figs. 14, 16). Robotic technology may offer solutions for safer, more reliable anastomotic techniques [40, 72, 73]. For neoadjuvant regimens, a general consensus exists [24, 29], though controversy persists [30]. Immunotherapy regimens are also being introduced in this setting [32].

Lymphadenectomy ultimately involves a balance between survival benefit and postoperative complications, rather than being simply assessed by the number of lymph nodes retrieved [74]. While this theme may regain research momentum driven by advancements in minimally invasive techniques—such as the enhanced visualization and superior dexterity afforded by RAMIE [75, 76]—and by the growing focus on lymph node pathological response following neoadjuvant therapy [77]. However, given that lymphadenectomy techniques have become largely standardized and that data from major clinical trials are now entering a mature phase, a short-term lack of breakthrough findings is expected [78, 79]. Consequently, the trajectory of this theme reflects a balance between renewed potential from technological innovation and a temporary plateau in evidence generation. Ultimately, the paramount research focus—and the most active theme (Fig. 16)—is the evaluation of fundamental outcomes (safety, complications, survival, and quality of life), serving as the gold standard for assessing surgical techniques and treatment paradigms. This is the domain of high-impact journals, highly cited papers, and leading research groups. Providing definitive answers requires large-scale, long-term studies that exceed the capacity of individual centers, underscoring the necessity for expansive national and international collaboration. Additional metrics like operative time, learning curves, and cost-effectiveness may also gain importance as the field evolves.

Based on the comprehensive analysis above, several key future directions emerge. The most immediate breakthrough may arise from large-scale clinical trials investigating neoadjuvant chemoimmunotherapy. Regarding surgical techniques, minimally invasive approaches have been firmly established as superior to open surgery. However, the optimal choice among specific procedures—whether McKeown, Ivor Lewis, or transmediastinal esophagectomy; conventional thoracoscopic-laparoscopic instruments versus robotic-assisted thoracic surgery (RATS); single-port versus multi-port approaches—remains to be determined. While patient selection based on preoperative tumor histology, location, and lymph node status may inform these decisions, more critically, definitive answers will require the accumulation of robust data through large-scale, multi-institutional and international collaboration. Such collaboration is essential to detect potentially subtle differences in complications, survival, and quality of life—differences that may be difficult to establish with statistical significance in smaller, single-center cohorts. Among these surgical innovations, RAMIE represents a particularly promising breakthrough. However, its widespread adoption hinges not only on continued technological refinement but also on meaningful reductions in equipment costs.

## Limitations

This study has several limitations. First, our search was confined to the Web of Science Core Collection, potentially missing relevant studies indexed elsewhere. Second, restricting to English-language publications may have introduced selection bias; despite the high publication output from non-English-speaking countries such as China, Japan, and the Netherlands—as demonstrated by our findings—potentially valuable clinical insights published in their native languages could have been overlooked. Third, very recent publications were excluded as they lack sufficient citation data for meaningful bibliometric analysis.

## Conclusion

The field of MIE has seen the thoracoscopic-laparoscopic approach become standard. Robotic assistance is now a key driver of innovation and a major research hotspot. For ESCC, immunotherapy is revolutionizing neoadjuvant treatment paradigms. Advancing the field definitively, for both techniques and systemic therapies, hinges on large-scale clinical validation with long-term follow-up, demanding extensive multi-institutional and international cooperation.

## Appendix

1: (TI = (“Esophagectomy” OR “Esophagectomies”) OR AB = (“Esophagectomy” OR “Esophagectomies”) OR AK = (“Esophagectomy” OR “Esophagectomies”).

2: (TI = (“Minimally Invasive Surgical Procedures” OR “Minimal Surgical Procedure” OR “Minimal Surgical Procedures” OR “Minimally Invasive Surgery” OR “Minimally Invasive Surgeries” OR “Surgeries, Minimally Invasive” OR “Surgery, Minimally Invasive” OR “Procedure, Minimal Surgical” OR “Procedures, Minimal Access Surgical” OR “Procedures, Minimal Surgical” OR “Procedures, Minimally Invasive Surgical” OR “Minimally Invasive Surgical Procedure” OR “Surgical Procedure, Minimal” OR “Surgical Procedures, Minimal” OR “Surgical Procedures, Minimal Access” OR “Minimal Access Surgical Procedures” OR “Surgical Procedures, Minimally Invasive” OR “minimally invasive” OR “inflatable mediastinoscopy” OR “inflatable video-assisted mediastinoscopic transhiatal” OR “robot-assisted”) OR AB = (“Minimally Invasive Surgical Procedures” OR “Minimal Surgical Procedure” OR “Minimal Surgical Procedures” OR “Minimally Invasive Surgery” OR “Minimally Invasive Surgeries” OR “Surgeries, Minimally Invasive” OR “Surgery, Minimally Invasive” OR “Procedure, Minimal Surgical” OR “Procedures, Minimal Access Surgical” OR “Procedures, Minimal Surgical” OR “Procedures, Minimally Invasive Surgical” OR “Minimally Invasive Surgical Procedure” OR “Surgical Procedure, Minimal” OR “Surgical Procedures, Minimal” OR “Surgical Procedures, Minimal Access” OR “Minimal Access Surgical Procedures” OR “Surgical Procedures, Minimally Invasive” OR “minimally invasive” OR “inflatable mediastinoscopy” OR “inflatable video-assisted mediastinoscopic transhiatal” OR “robot-assisted”) OR AK = (“Minimally Invasive Surgical Procedures” OR “Minimal Surgical Procedure” OR “Minimal Surgical Procedures” OR “Minimally Invasive Surgery” OR “Minimally Invasive Surgeries” OR “Surgeries, Minimally Invasive” OR “Surgery, Minimally Invasive” OR “Procedure, Minimal Surgical” OR “Procedures, Minimal Access Surgical” OR “Procedures, Minimal Surgical” OR “Procedures, Minimally Invasive Surgical” OR “Minimally Invasive Surgical Procedure” OR “Surgical Procedure, Minimal” OR “Surgical Procedures, Minimal” OR “Surgical Procedures, Minimal Access” OR “Minimal Access Surgical Procedures” OR “Surgical Procedures, Minimally Invasive” OR “minimally invasive” OR “inflatable mediastinoscopy” OR “inflatable video-assisted mediastinoscopic transhiatal” OR “robot-assisted”).

3: (TI = (“Thoracoscopic Laparoscopic Esophagectomy” OR “Thoracoscopic Esophagectomy” OR “Laparoscopic Esophagectomy”) OR AB = (“Thoracoscopic Laparoscopic Esophagectomy” OR “Thoracoscopic Esophagectomy” OR “Laparoscopic Esophagectomy”) OR AK = (“Thoracoscopic Laparoscopic Esophagectomy” OR “Thoracoscopic Esophagectomy” OR “Laparoscopic Esophagectomy”).

4: (TI = (“Cancer of Esophagus” OR “Esophageal Cancer” OR “Cancer, Esophageal” OR “Cancers, Esophageal” OR “Esophageal Cancers” OR “Cancer of the Esophagus” OR “Esophagus Cancer” OR “Cancer, Esophagus” OR “Cancers, Esophagus” OR “Esophagus Cancers” OR “cancer” OR “carcinoma”) OR AB = (“Cancer of Esophagus” OR “Esophageal Cancer” OR “Cancer, Esophageal” OR “Cancers, Esophageal” OR “Esophageal Cancers” OR “Cancer of the Esophagus” OR “Esophagus Cancer” OR “Cancer, Esophagus” OR “Cancers, Esophagus” OR “Esophagus Cancers” OR “cancer” OR “carcinoma”) OR AK = (“Cancer of Esophagus” OR “Esophageal Cancer” OR “Cancer, Esophageal” OR “Cancers, Esophageal” OR “Esophageal Cancers” OR “Cancer of the Esophagus” OR “Esophagus Cancer” OR “Cancer, Esophagus” OR “Cancers, Esophagus” OR “Esophagus Cancers” OR “cancer” OR “carcinoma”).

5: ((#1 AND #2) OR #3) AND #4).
